# Treatment of relapsed/refractory chronic lymphocytic leukemia/small lymphocytic lymphoma with the BTK inhibitor zanubrutinib: phase 2, single-arm, multicenter study

**DOI:** 10.1186/s13045-020-00884-4

**Published:** 2020-05-11

**Authors:** Wei Xu, Shenmiao Yang, Keshu Zhou, Ling Pan, Zengjun Li, Jianfeng Zhou, Sujun Gao, Daobin Zhou, Jianda Hu, Ru Feng, Haiwen Huang, Meng Ji, Haiyi Guo, Jane Huang, William Novotny, Shibao Feng, Jianyong Li

**Affiliations:** 1 Department of Hematology, Jiangsu Province Hospital, Collaborative Innovation Center for Cancer Personalized Medicine, The First Affiliated Hospital of Nanjing Medical University, 300 Guangzhou Road, Nanjing, 210029 Jiangsu China; 2 Peking University Peoples Hospital, Peking University Institute of Hematology, Beijing, China; 3 Henan Cancer Hospital, Affiliated Cancer Hospital of Zhengzhou University, Zhengzhou, China; 4 West China Hospital of Sichuan University, Chengdu, China; 5 Blood Disease Hospital, Chinese Academy of Medical Science, Tianjin, China; 6 Tongji Hospital, Tongji Medical College, Wuhan, China; 7 The First Hospital of Jilin University, Changchun, China; 8 Peking Union Medical College Hospital, Chinese Academy of Medical Sciences & Peking Union Medical College, Beijing, China; 9 Fujian Institute of Hematology, Fujian Provincial Key Laboratory of Hematology, Fujian Medical University Union Hospital, Fuzhou, China; 10 Nanfang Hospital of Southern Medical University, Guangzhou, China; 11 The 1st Hospital of Soochow University, Suzhou, China; 12 BeiGene (Beijing) Co., Ltd., Beijing, China; 13 BeiGene USA, Inc., San Mateo, CA USA

**Keywords:** Bruton’s tyrosine kinase, Chronic lymphocytic leukemia, Relapsed/refractory, Zanubrutinib, Clinical trial

## Abstract

**Background:**

Bruton tyrosine kinase (BTK) inhibitors have demonstrated a high degree of efficacy in the treatment of B cell malignancies characterized by constitutive B cell receptor activation, including chronic lymphocytic leukemia/small lymphocytic lymphoma (CLL/SLL).

**Methods:**

The efficacy and safety of zanubrutinib, an investigational highly selective BTK inhibitor, was evaluated in this single-arm, phase 2 study of Chinese patients with relapsed/refractory CLL/SLL. The primary endpoint was overall response rate as assessed by an independent review committee.

**Results:**

Of the 91 evaluable patients, 77 (84.6%) achieved a response, with three (3.3%), 54 (59.3%), and 20 (22%) patients achieving a complete response, partial response, and partial response with lymphocytosis, respectively, after a median follow-up of 15.1 months. The estimated 12-month event-free rate for duration of response was 92.9%. The most commonly reported grade ≥ 3 adverse events (AEs) were neutropenia (44%), thrombocytopenia (15.4%), lung infection/pneumonia (13.2%), upper respiratory tract infection (9.9%), and anemia (8.8%). The 12-month overall survival rate was 96%. Eight (9.0%) patients discontinued zanubrutinib due to AEs, and seven (8.0%) patients required at least one dose reduction.

**Conclusion:**

Treatment of patients with relapsed/refractory CLL/SLL with zanubrutinib was generally well tolerated and resulted in a high overall response rate, thereby conferring a favorable benefit-risk profile.

**Trial registration:**

Prospectively registered in China public registry (CTR20160890) on December 7, 2016: http://www.chinadrugtrials.org.cn/. Retrospectively registered in ClinicalTrials.gov (NCT03206918) on July 2, 2017.

## Background

Advances in the development of novel targeted agents have significantly improved outcomes for patients with chronic lymphocytic leukemia/small lymphocytic lymphoma (CLL/SLL), particularly those who have either failed or are not candidates for standard immunochemotherapy regimens and/or have high-risk prognostic features (e.g., del[17p], del[11q], complex karyotype cytogenetics, and unmutated immunoglobulin heavy-chain variable region [IGHV]). Principal among these are the inhibitor of phosphoinositide triphosphate kinase delta isoform, idelalisib [1], the B cell lymphoma-2 homology 3 mimetic, venetoclax [2], and the first-in-class Bruton tyrosine kinase (BTK) inhibitor ibrutinib [3].

Constitutive B cell receptor signaling is an important contributor to the survival and proliferation of CLL/SLL [4, 5]. Inhibition of BTK and other proximal kinases in the B cell receptor signaling pathway has been shown to induce profound inhibition of proliferative signaling stemming from the interactions of leukemic cells and their microenvironment [6–8]. In preclinical studies, ibrutinib inhibited malignant B cell proliferation and survival in vivo, as well as cell migration and substrate adhesion in vitro. Based on the demonstration of clinical benefit in phase 3 studies of relapsed or refractory [9] and treatment-naïve [10] CLL/SLL patients, ibrutinib was granted approval by the US Food and Drug Administration for the treatment of patients with CLL/SLL with or without 17p deletion [11].

Despite the relative clinical success of ibrutinib in the management of CLL/SLL, it is not curative, and complete responses (CR) are relatively uncommon with single-agent therapy. The frequency of adverse events (AE) is also treatment-limiting, resulting in the need for discontinuation in 21% of relapsed/refractory clinical trial participants after a median treatment duration of 39 months [12]; in a clinical practice setting, the discontinuation rate among patients with relapsed/refractory CLL is reported to be as high as 50% [13]. The most frequent AEs leading to discontinuation of ibrutinib include arthralgia, atrial fibrillation, bleeding, second malignancy, general debility, infection, and pneumonitis [14].

Zanubrutinib (BGB-3111) is a novel, oral BTK inhibitor which, like other active BTK inhibitors, forms an irreversible covalent bond at Cys_481_ within the adenosine triphosphate binding pocket of BTK. Zanubrutinib is highly potent against BTK (IC_50_, 0.3 nM). Compared with ibrutinib, it exhibited less off-target inhibition of other tyrosine kinases, such as EGFR, JAK3, TEC, ITK, and others, in both kinase inhibition and cell-based assays [15]. Off-target kinase inhibition is thought to mediate ibrutinib-associated toxicities, such as diarrhea and rash (associated with EGFR inhibition) [3, 9, 10], bleeding or bruising [16, 17], and atrial fibrillation [18, 19]. In two phase 1/2 studies conducted within (BGB-3111-1002 [NCT03189524]) and outside (BGB-3111-AU-003 [NCT02343120]) of China, zanubrutinib demonstrated generally good tolerability without dose-limiting toxicities at daily doses up to 320 mg, the highest dose tested in both studies. The recommended phase 2 dose was determined to be 160 mg administered twice daily by mouth, based on pharmacokinetic, pharmacodynamic, preliminary safety, and efficacy results in patients with a variety of B cell malignancies [15, 45]. Based on promising preliminary results in the BGB-3111-AU-003 study, including an overall response rate (ORR) of 96% in patients with CLL/SLL [15], we initiated this phase 2 trial of zanubrutinib in Chinese patients with relapsed/refractory CLL/SLL.

## Methods

### Patients

Patients were enrolled at 11 clinical trial sites in China between March 9 and December 14, 2017. Eligible patients had CLL or SLL, as defined by International Workshop on Chronic Lymphocytic Leukemia [20] or World Health Organization criteria [21], respectively, and that was histologically confirmed by central pathologic review; measurable disease; and met requirements for treatment [20, 22]. Patients must have had relapsed/refractory disease after a minimum of one prior line of a standard chemotherapy regimen (e.g., fludarabine or chlorambucil-based) administered over at least two cycles. Other eligibility criteria included age ≥ 18 years, Eastern Cooperative Oncology Group performance status ≤ 2, and adequate renal (creatinine clearance ≥ 30 mL/min) and liver function (aspartate and aminotransferase levels ≤ 3× upper limit of normal [ULN], bilirubin ≤ 2× ULN). Patients had to have an absolute neutrophil count ≥ 0.75 × 10^9^/L and a platelet count ≥ 50 × 10^9^/L, independent of growth factor support or transfusion, respectively, within 7 days of study entry. Patients were excluded if they had a history or evidence of central nervous system lymphoma; prior exposure to a BTK inhibitor; clinically significant cardiovascular disease or a myocardial infarction, cerebrovascular accident, or intracranial hemorrhage within the previous 6 months; prior allogeneic hematopoietic stem cell transplant (relapsed patients after at least 6 months following an autologous transplant were eligible); known infection with HIV or serologic status reflecting active hepatitis B virus (HBV) or hepatitis C virus (HCV) infection; or known or suspected Richter transformation. Patients who were seropositive for HBV core antibody but HBV DNA negative at baseline were eligible if they consented to monthly HBV DNA monitoring or preemptive antiviral prophylaxis. In a subsequent amendment to the study protocol, all patients at risk for HBV reactivation were required to undergo both monthly testing for HBV DNA and antiviral prophylaxis. There was no exclusion for patients who were receiving either concurrent antacids (including proton-pump inhibitors [PPIs]) or antithrombotic agents. Concurrent use of strong CYP3A inhibitors and strong CYP3A inducers was to be avoided. Strong CYP3A inhibitors (e.g., antimycotic agents and antibiotics) were permitted only if no other treatment alternative was available during which dose interruption of zanubrutinib was to be considered. Transfusion and myeloid growth factor support (e.g., granulocyte colony stimulating factor [G-CSF]) were permitted for patients with peripheral blood cytopenias.

### Study design and treatment

This is an ongoing phase 2, open-label, single-arm study of zanubrutinib in Chinese patients with relapsed/refractory CLL/SLL. All patients received zanubrutinib 160 mg twice daily in 28-day cycles until disease progression or intolerance. This study was designed and monitored in accordance with sponsor procedures and in compliance with the ethical principles of Good Clinical Practice, International Conference on Harmonization guidelines, the Declaration of Helsinki, and applicable local regulatory requirements. All patients provided written informed consent. The protocol, any amendments, and informed consent forms were approved by the institutional review boards/independent ethics committees. The study is registered at ClinicalTrials.gov (NCT03206918).

### Assessments

The primary endpoint was ORR as assessed by an independent review committee (IRC) (PARAXEL Informatics, Waltham, MA) in accordance with published guidelines [20, 22]. Qualifying responses for patients with CLL included partial response (PR) with lymphocytosis, nodular PR, PR, CR, or CR with incomplete hematologic recovery and for patients with SLL, either PR or CR. Secondary endpoints included IRC-assessed progression-free survival (PFS), duration of response (DOR), time to response, investigator-assessed ORR, and safety. Response evaluations were based on clinical and radiographic assessments. All patients underwent contrast-enhanced CT (or MRI) scans every 12 weeks for the first 48 weeks of the study and then every 24 weeks thereafter until progression or study termination. Patients underwent bone marrow aspiration and biopsy at baseline and for confirmation of CR or if disease progression was suspected due to worsening peripheral blood cytopenias. Standard laboratory assessments for safety monitoring were performed coincident with each study visit and at the end of treatment; ECGs were evaluated at baseline, end of treatment, and at other times as clinically indicated. AEs were coded using the Medical Dictionary for Regulatory Activities (MedDRA), version 20.0 [23] and graded for severity based on National Cancer Institute Common Toxicity Criteria (NCI CTCAE), version 4.03 [24]. All treatment-emergent AEs, including AEs of interest (based on the known toxicity profile for BTK inhibitors) that occurred on or after the first treatment day until 30 days after study treatment discontinuation were summarized. Each category of AEs of interest included event terms identified in accordance with predefined MedDRA search criteria as outlined in Supplemental Table 1. Patients who discontinued study treatment for reasons other than progressive disease (PD) continued to undergo disease assessments until progression or withdrawal of consent.

### Statistical analysis

Sample size considerations were based on the level of precision of the estimated ORR and power of its comparison with historical data. With 80 patients and assuming an ORR of 63% for zanubrutinib versus an ORR of 32% in historical controls [25] and using a binomial exact test, the power was greater than 0.99 to demonstrate statistical significance at a one-sided alpha of 0.025. Primary efficacy and safety analyses included all patients with centrally confirmed CLL/SLL who received at least one dose of zanubrutinib. Overall response rate was summarized as the percentage of responders with 95% confidence intervals [26]. Subgroup analysis for prespecified demographic and baseline disease characteristics was conducted for ORR. Duration of response was assessed from the time of the first response until PD or death from any cause. Progression-free survival was measured from the time of first dose to PD or death from any cause. Patients who did not experience PD or death were censored on the day of the last tumor assessment before initiation of subsequent anticancer therapy for analyses of DOR and PFS. Median DOR and PFS as well as event-free rates at landmark timepoints were estimated using Kaplan-Meier methodology with corresponding 95% confidence intervals [27, 28]. Follow-up times for PFS and DOR were estimated by the reverse Kaplan-Meier method.

## Results

### Patients

Ninety-one patients (82 CLL, 9 SLL) enrolled in the study and received at least one dose of zanubrutinib. The median number of 28-day cycles was 15 (range, 1–24 cycles). All enrolled patients were evaluable for safety and efficacy. Baseline demographic and disease characteristics for the study population are summarized in Table 1. Most patients (69.2%) had advanced clinical stage (Binet stage C CLL or stage III/IV SLL) and at least one additional poor prognostic variable, including unmutated IGHV (56.0%), del(17p) or *TP53* mutation (24.2%), and/or del(11q) (22%). Approximately half (49.5%) of the patients had received two or more prior lines of therapy, and most (79.1%) were refractory to their most recent therapy.

**Table 1:** Baseline demographic and disease characteristics

Baseline characteristics	*N* = 91
**Male,*n*(%)**	52 (57.1)
**Median age (range), years**	61.0 (35–87)
**ECOG PS,*n*(%)**	
0/1	88 (96.7)
2	3 (3.3)
**CLL,*n*(%)**	82 (90.1)
**SLL,*n*(%)**	9 (9.9)
**Median time since initial diagnosis, months (range)**	39.4 (3.2–185.1)
**Number of prior lines of therapy**	
Median	1.0
Min, max	1, 9
≥ 2 prior lines of therapy, *n* (%)	45 (49.5)
**Bulky disease,**^**a**^***n*(%)**	40 (44.0)
**Binet stage at study entry for CLL patients**^**b**^**,*n*(%)**	
Stage A/B	27 (32.9)
Stage C	55 (67.1)
**Ann Arbor stage at study entry for SLL patients,*n*(%)**	
Stage I	1 (11.1)
Stage III/IV	8 (88.9)
**β2 microglobulin > 3.5 mg/L,*n*(%)**	68 (74.7)
**Unmutated IGHV*,***^**c**^***n*(%)**	51 (56.0)
**Del(17p) or*TP53*mutation,*n*(%)**	22 (24.2)
**Del(13q),*n*(%)**	41 (45.1)
**Del(11q),*n*(%)**	20 (22.0)
**Prior anticancer drug therapy,*n*(%)**^**d**^	
Alkylating agents (including bendamustine)	68 (74.7)
Nucleoside analogs	52 (57.1)
Anti-CD20-based therapy	54 (59.3)
Anti-CD20-based chemoimmunotherapy	44 (48.4)
Lenalidomide/thalidomide	7 (7.7)
Other therapies	12 (13.2)
**Refractory to last systemic therapy,*n*(%)**	72 (79.1)

*CLL* chronic lymphocytic leukemia, *ECOG PS* Eastern Cooperative Oncology Group performance status, *IGHV* immunoglobulin heavy-chain variable region, *LDi* longest diameter, *max* maximum, *min* minimum, *SLL* small lymphocytic lymphoma

^a^Bulky disease refers to ≥ 1 lesion with LDi ≥ 5 cm

^b^*n* = 82

^c^The IGHV mutational status was unknown in 17 patients for the following reasons: IGHV gene rearrangement undetected (three patients); multiclonal IGHV gene rearrangement detected (13 patients); test failed (one patient)

^d^Nucleoside analog is defined as any regimen that includes fludarabine; alkylating agent is defined as any regimen that includes an alkylator without fludarabine; anti-CD20-based therapy is defined as any regimen that includes rituximab either alone or with other regimen components; anti-CD20-based chemoimmunotherapy is defined as any regimen that includes both rituximab and cytotoxic agents. Other includes VDAE (vindesine, methylprednisolone, pirarubicin, and etoposide), DEMP (vindesine, methylprednisolone, mitoxantrone, and etoposide), ESHAP (etoposide, cisplatin, cytarabine, with or without mercaptopurine or prednisone), GP (gemcitabine and oxaliplatin), anti-CD52 monoclonal antibody, methylprednisolone only, cisplatin and dendritic cell-activated, cytokine-induced killer cells (DCCIK), and interferon only. The categories are not mutually exclusive

After a median follow-up of 15.1 months (range, 0.8 to 21.2 months), 16 (17.6%) patients discontinued zanubrutinib (6 due to PD, 1 due to Richter transformation, 8 due to AEs, and 1 after withdrawal of consent). A total of 85 patients (93.4%) were continuing in the study; six (6.6%) discontinued study participation due to death (*n* = 4) or withdrawal of consent (*n* = 2).

### Efficacy

**Fig. 1:**
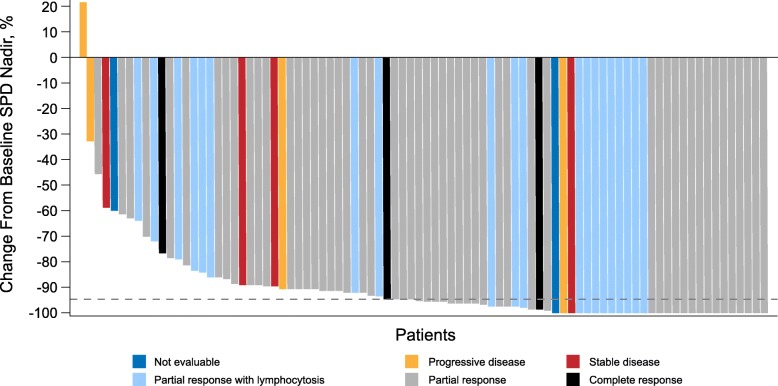
Maximal Percent Change in the Sum of the Products of Perpendicular Diameters of Target Lesions (SPD) and Corresponding Best Responses

**Fig. 2:**
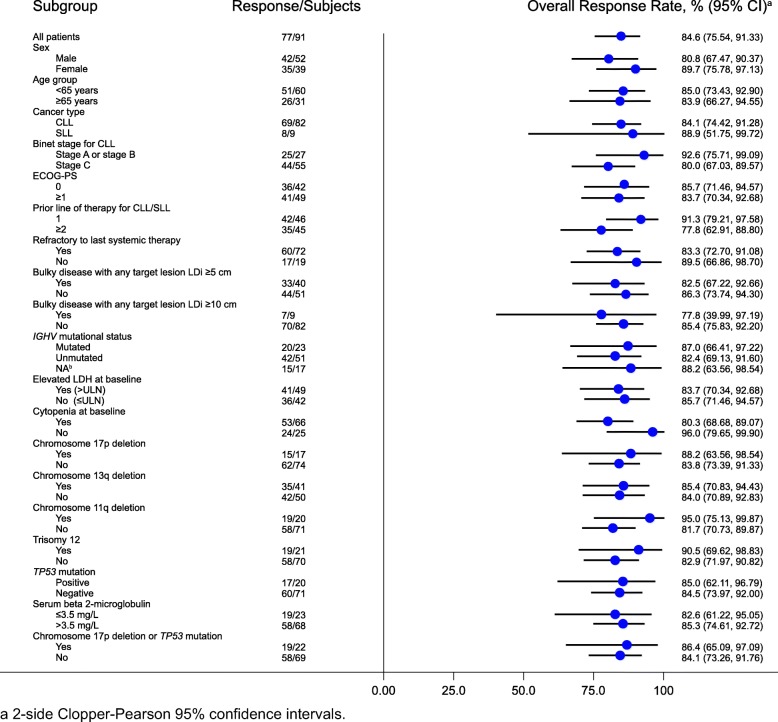
Subgroup analysis of ORR

**Fig. 3:**
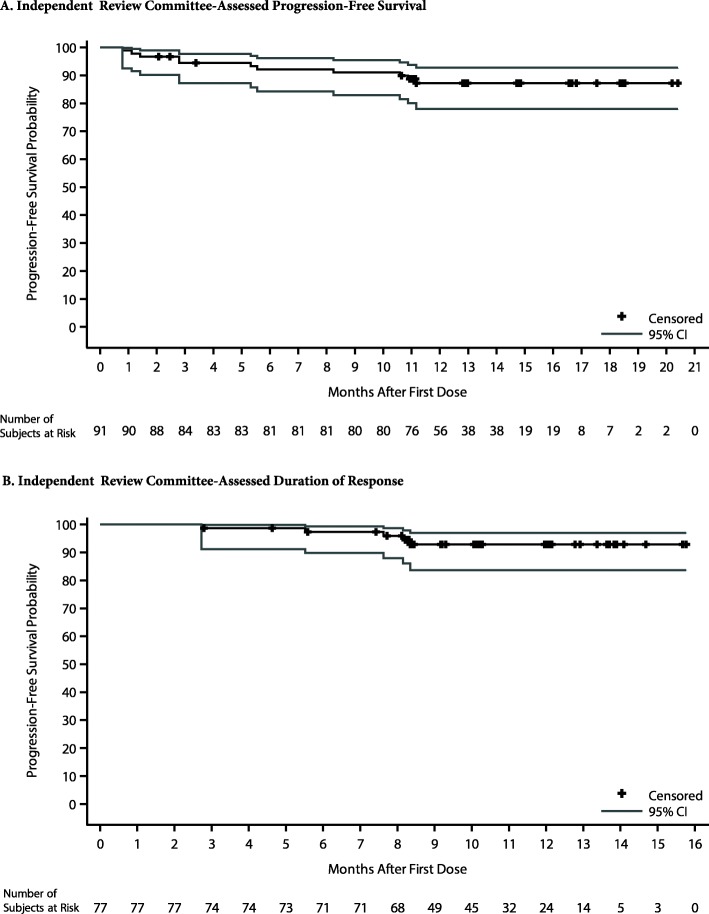
**a** Independent review committee-assessed progression-free survival. **b** Independent review committee-assessed duration of response

A total of 77 (84.6%, 95% CI, 75.5–91.3) relapsed/refractory patients achieved a response, including 69 with CLL and eight with SLL (*P <* 0.0001 with respect to the null hypothesis of an ORR of 32%). Fifty-seven (62.6%) patients achieved a PR or better and an additional 20 (22%) achieved a best response of PR with lymphocytosis. All three patients who achieved a CR had SLL (Table 2). All except one patient exhibited reductions in tumor burden, most by ≥ 50% (Fig. 1). Subgroup analysis of ORR revealed results generally consistent with the overall study population, including in subgroups with poor prognostic features (e.g., IGHV unmutated status [82%], del(17p)/*TP53* mutation [86%], and refractory disease [83%]); Fig. 2). The median time to onset of response was 2.8 months (25th–75th percentile, 2.8–2.9); 64 (83%) patients achieved a response by the first assessment timepoint. The concordance rate between IRC- and investigator-assessed response was 79.1% for best response achieved, 87.9% for patients with a best response of PR or better, and 91.2% for response overall. After median follow-up of 12.9 months (range, 0.8–20.4 months) for PFS, an estimated 87.2% of patients had neither progressed nor died at 12 months; the median PFS has not been reached (Table 3; Fig. 3a). Five of 77 responders progressed from 2.7 to 8.3 months after initial response, while an estimated 92.9% of responders were event-free at 12 months (Table 3; Fig. 3b). As of the data cutoff date, four patients died, all within 30 days of last study treatment (2 from complications of PD and 2 from AEs) for a 12-month estimated overall survival rate of 95.6%.

**Table 2:** Independent review committee-assessed efficacy outcomes

Efficacy variable	*N* = 91
**Objective response,*n*(%)**	
CR	3 (3.3)
CRi	0 (0.0)
nPR	0 (0.0)
PR	54 (59.3)
PR-L	20 (22.0)
No response^a^	8 (8.8)
Not evaluable^b^	6 (6.6)
Overall (%)	84.6
95% CI for overall response rate	75.5, 91.3
*P* value^c^	< 0.0001
**Time to response,**^**d**^**months**	
Median (range)	2.8 (2.6–8.4)
**Duration of response, months**	
Median^e^ (range)	NE
95% CI	NE, NE
Event-free rates^f^ at 12 months (%)	92.9
95% CI	83.6, 97.0
**Progression-free survival, months**	
Median^e^ (range)	NE
95% CI	NE, NE
Event-free rates^f^ at 12 months (%)	87.2
95% CI	78.0, 92.7

Efficacy outcomes were assessed in accordance with International Workshop on Chronic Lymphocytic Leukemia guidelines [20] for CLL patients and the Lugano classification [22] for SLL patients

*CR* complete response, *CRi* complete response with incomplete bone marrow recovery, *NE* not estimable, *nPR* nodular partial response, *ORR* overall response rate, *PR* partial response, *PR-L* partial response with lymphocytosis

^a^Includes all patients with a best overall response of stable disease or progressive disease

^b^Two patients had incomplete imaging studies; one patient had only one response assessment without evidence of response maintenance for at least 2 months; three patients discontinued prior to first post-baseline assessment

^c^One-sided *P* value was based on exact test comparison of zanubrutinib ORR versus reference rate (H_0_) of 0.32

^d^Summarized for patients who achieved a response of PR-L or better

^e^Medians were estimated by Kaplan-Meier methodology with 95% confidence intervals estimated using the Brookmeyer and Crowley method [27]. NE denotes not estimable

^f^Event-free rates were estimated by Kaplan-Meier methodology with 95% confidence intervals estimated using Greenwood’s formula [28]

**Table 3:** Adverse events

Term	All grades	Grade 3	Grade 4
	*n* (%)		
Patients with at least 1 adverse event	91 (100)	58 (63.7)	8 (8.8)
**Hematologic events**			
Neutropenia^a^	63 (69.2)	34 (37.4)	6 (6.6)
Thrombocytopenia^b^	38 (41.8)	13 (14.3)	1 (1.1)
Anemia^c^	27 (29.7)	8 (8.8)	0
Leukopenia^d^	19 (20.9)	6 (6.6)	0
**Nonhematologic events**			
Upper respiratory tract infection	41 (45.1)	9 (9.9)	0
Hematuria/blood urine present	36 (39.6)	0	0
Petechiae, purpura, or contusion	32 (35.2)	0	0
Hypokalemia	23 (25.3)	6 (6.6)	0
Cough	22 (24.2)	1 (1.1)	0
Carbon dioxide increased	19 (20.9)	0	0
Hyperglycemia	19 (20.9)	2 (2.2)	0
Diarrhea	18 (19.8)	2 (2.2)	0
Lung infection/pneumonia	20 (22.0)	11 (12.1)	0^e^
Urinary tract infection	15 (16.5)	1 (1.1)	0
Rash	13 (14.3)	0	0
Urobilinogen increased	12 (13.2)	0	0
Alanine amino transferase increased	11 (12.1)	1 (1.1)	0
Hypoalbuminemia	11 (12.1)	1 (1.1)	0
Aspartate amino transferase increased	10 (11.0)	1 (1.1)	0
Hyperuricemia	10 (11.0)	1 (1.1)	1 (1.1)
Pyrexia	10 (11.0)	0	0
Hyponatremia	9 (9.9)	4 (4.4)	0
Hypocalcemia	9 (9.9)	2 (2.2)	0
Hypertension^f^	9 (9.9)	2 (2.2)	0
Skin infection	8 (8.8)	2 (2.2)	0
Bronchitis	6 (6.6)	2 (2.2)	0
Gastroenteritis	5 (5.5)	2 (2.2)	0
Hepatitis B reactivation	3 (3.3)	2 (2.2)	0
Infectious enteritis	2 (2.2)	2 (2.2)	0

Data are for treatment-emergent adverse events in the 91 patients included in the study. Listed events occurred in at least 10% of patients, or for grade ≥ 3 in at least 2% of patients

*MedDRA* Medical Dictionary for Regulatory Activities

^a^Includes the MedDRA preferred terms neutropenia and neutrophil count decreased

^b^Includes the MedDRA preferred terms thrombocytopenia and platelet count decreased

^c^Includes the MedDRA preferred terms anemia and hemoglobin decreased

^d^Includes the MedDRA preferred term white blood cell count decreased

^e^One patient had a grade 5 lung infection (see text)

^f^Includes the MedDRA preferred terms hypertension and blood pressure increased

### Safety

All patients reported at least one AE. Fifty-eight (63.7%) patients reported ≥1 grade 3 AE, eight (8.8%) patients reported grade 4 AEs, and three (3.3%) had grade 5 events (one in the setting of PD). The most frequently reported AEs of any grade were neutropenia (69.2%), upper respiratory tract infection (45.1%), thrombocytopenia (41.8%), petechiae/purpura/contusion (35.2%), anemia and hematuria (each 29.7%), hypokalemia (25.3%), cough (24.2%), and increased carbon dioxide and hyperglycemia (each 20.9%). The most common grade 3 AEs were neutropenia (37.4%), thrombocytopenia (14.3%), lung infection/pneumonia (12.1%), upper respiratory tract infection (9.9%), and anemia (8.8%). Six patients reported grade 4 neutropenia, one reported grade 4 thrombocytopenia, and one reported grade 4 hyperuricemia, the latter without other laboratory or clinical manifestations of tumor lysis syndrome. Fifteen (16.5%) patients had baseline absolute neutrophil counts < 1.5 × 10^9^/L. A total of 32 (35.2%) patients received at least one course of G-CSF (filgrastim, or nartograstim) or granulocyte-macrophage colony-stimulating factor (molgramostim). One third of patients reported at least one serious AE, the most common being lung infection (*n* = 7), pneumonia (*n* = 3), upper respiratory infection (*n* = 3), and bronchitis (*n* = 2). Three patients had grade 5 (fatal) events: lung infection accompanied by cardiac and respiratory failure on study day 24 in a 66-year-old male with one prior regimen for CLL, who received 6 days of study drug; cardiopulmonary failure on study day 43 in a 67-year-old male with three prior regimens for CLL in the setting of high tumor burden and chronic obstructive pulmonary disease; and multiorgan system failure in the setting of PD on study day 46 in a 52-year-old male with nine prior regimens for CLL. The latter patient had extensive disease at baseline (serum lactate dehydrogenase 565 U/L and bulky adenopathy), but was without other evidence of Richter transformation. Eight (9%) patients discontinued zanubrutinib due to AEs, and seven (8.0%) patients required at least one dose reduction (Supplemental Table 2). Among the AEs of interest, the most common grade ≥ 3 events were in the categories of infection (38.5%), neutropenia (44%), thrombocytopenia (15.4%), and anemia (8.8%) (Supplemental Table 3). Most infections were mucosal infections of the respiratory or urinary tracts. In addition to the relatively frequent occurrence of cutaneous bleeding noted above, hematuria and/or blood urine present was reported in 36 patients, subcutaneous bleeding in 6 patients, and epistaxis in 5; all were grade 1 or 2 events. Two patients experienced major hemorrhagic events: a gastrointestinal hemorrhage in association with newly diagnosed colon cancer in 1 patient, and the second, a right-sided thalamic hemorrhage after a fall, both assessed as unrelated to study drug. Two (3.0%) patients had treatment-emergent grade 3 hypertension, neither of which led to treatment modification, and two (3.0%) had second primary malignancies (one with newly diagnosed breast cancer and the aforementioned patient with colon cancer). No patient developed atrial fibrillation or flutter, and there were no cases of tumor lysis syndrome. Transient increases in peripheral blood lymphocyte counts observed beginning in cycle 2 are consistent with the propensity for BTK inhibitors to stimulate migration of tumor cells from the lymphoid compartment into the blood (Supplemental Figure 1) [3, 29].

A total of 27 (29.7%) patients had serologic evidence of prior HBV infection at baseline. Of these, four patients exhibited findings consistent with HBV reactivation in the setting of protocol-specified serologic surveillance for the latter; none of these patients were receiving antiviral prophylaxis at the time of viral reactivation. No patient had symptoms coincident with HBV reactivation, and all events resolved uneventfully with a combination of antiviral therapy (*n* = 3) and/or zanubrutinib treatment modification (*n* = 3). One patient with grade 1 HBV reactivation exhibited low levels of HBV DNA in plasma and mild, transient elevation of serum bilirubin with no other abnormalities of liver function, which resolved within 28 days of onset without the need for antiviral therapy or study treatment modification. One additional patient experienced an apparent acute HBV infection while on treatment that responded to antiviral therapy in conjunction with zanubrutinib discontinuation.

## Discussion

Ibrutinib has demonstrated clinically meaningful activity in patients with relapsed/refractory CLL/SLL and has become a standard of care for patients with CLL/SLL, both in the frontline (especially those who are not candidates for immunochemotherapy) and relapsed/refractory settings. Toxicities, some likely related to off-target effects, are relatively frequent causes for ibrutinib discontinuation both in clinical trial [12] and clinical practice settings [13]. Zanubrutinib has shown improved specificity for BTK compared with ibrutinib; at doses of 160 mg twice daily, zanubrutinib steady-state plasma levels were approximately eightfold higher (after adjusting for 94.2% plasma protein binding) compared with those observed for ibrutinib at a daily dose of 560 mg [30, 31]. No ethnic differences in the pharmacokinetics of zanubrutinib have been observed between Asian and non-Asian patients [32]. Similar to other BTK inhibitors, zanubrutinib is primarily metabolized by CYP3A. Concurrent administration with strong or moderate CYP3A inhibitors (e.g., some antimicrobial agents) has been shown to significantly increase exposure levels of ibrutinib in drug-drug interaction studies [11, 32, 34]. The impact of CYP3A inhibitors on the exposure of zanubrutinib (2.6-fold increase in maximal plasma concentration [*C*_max_] upon itraconazole coadministration) [32] is significantly lower than that noted for ibrutinib (6.7-fold and 29-fold increase in *C*_max_ upon voriconazole and ketoconazole coadministration, respectively) [33, 34]. In the current study, 1 patient exhibited grade 4 neutropenia 4 weeks after completion of an extended course of fluconazole (a moderate CYP3A inhibitor) while concurrently receiving full dose zanubrutinib; 6 additional patients treated with a concurrent strong or moderate CYP3A inhibitor reported neither grade ≥ 3 nor serious AEs from the time of antimycotic therapy initiation until 30 days after completion (Supplemental Table 4). Unlike acalabrutinib, zanubrutinib can be coadministered with gastric acid-reducing agents (including PPIs) without restriction, as these have not been shown to impact its absorption [35].

In the current study, Chinese patients with relapsed/refractory CLL/SLL achieved an ORR of 84.6%, as assessed by an IRC after a median study follow-up of 15.1 months. Responses were observed in 19 (86.4%) of 22 patients with either del(17p) or *TP53* mutation, 19 (95%) of 20 patients with del(11q), and 42 (82%) of 51 patients with unmutated IGHV. Consistent with previous experience with other BTK inhibitors, CRs were uncommon and restricted to patients with SLL [3, 9, 10, 29]. Efficacy results reported herein were generally consistent with those observed in a cohort of zanubrutinib-treated patients with CLL/SLL from clinical trial sites outside of China (Australia, New Zealand, South Korea, the USA, the UK, and Italy), who were enrolled in the phase 1/2 BGB-3111-AU-003 study [15]. Among 56 relapsed/refractory patients in this cohort, the ORR was 94.6% (one CR, 45 PRs, seven PRs with lymphocytosis); all response-evaluable patients with del(17p) (*n* = 16) achieved a response to zanubrutinib. At a median study follow-up of 13.7 months (range, 0.4–30.5 months), 94.7% of CLL/SLL patients in BGB-3111-AU-003 were continuing zanubrutinib treatment [15]. Responses to zanubrutinib in the current study were durable, with an estimated 93% of responders free from progression after a median follow-up of 10.2 months; to date, one patient has developed Richter transformation. Progression-free survival at 12 months was 87.2%. Longer follow-up is needed to more precisely define efficacy outcomes for this CLL/SLL cohort. Similar results have been reported in the RESONATE study of patients with R/R CLL/SLL comparing ibrutinib with the anti-CD20, ofatumumab: 90% of ibrutinib-treated patients achieved a response after a median follow-up of 19 months, based on investigator assessments, with 76% progression-free at 18 months [36]. Despite the similarity of outcomes between the RESONATE population and the current study, there were some notable differences in baseline characteristics: patients in the former study were older (median age, 67 vs 61 years, respectively) had more advanced disease (median time from initial diagnosis 92 vs 39 months, respectively) and were more heavily pretreated (median of 3 vs 1 prior regimens, respectively). The last consideration is particularly relevant given that ibrutinib-treated patients in RESONATE with only 1 prior regimen had superior outcomes both in terms of ORR and 18-month PFS compared with those with ≥ 2 prior regimens [36]. Response rates were also higher in the current study among patients with 1 vs ≥ 2 prior regimens (91.3% and 77.8%, respectively, Fig. 2). Among relapsed/refractory CLL patients, similar response rates to those for both ibrutinib and zanubrutinib have been reported with the second-generation BTK inhibitor acalabrutinib (81%-95%) [29, 37].

After a median exposure of 13.7 months, adverse events reported in the current study were mostly of mild or moderate severity and generally consistent with the known toxicity profile for BTK inhibitors as well as the natural history of relapsed/refractory CLL/SLL. Similar to ibrutinib clinical trials in this disease setting [3, 9, 38], infections figure prominently in the spectrum of AEs (Table 3; Supplemental Table 3) with 38.5% of patients having reported at least one grade ≥ 3 infection compared with 24 to 30% for ibrutinib [9, 38]. Predictably, these were mainly respiratory tract infections, both all grade and grade ≥ 3. In particular, the frequency of grade ≥ 3 lung infection/pneumonia reported in the current study (13.2%) is consistent with that reported among ibrutinib-treated patients both outside (12 to 13%) [9, 38] and within China (16.3%) [39]. Most infections were effectively managed with adequate resolution and without the need for dose reduction or treatment discontinuation.

Infectious complications are commonplace in CLL and account for 50 to 60% of CLL deaths [40]. Multiple etiologies including disease-related immuno- and myelosuppression as well as treatment with alkylating agents and corticosteroids are major contributors to the risk of primarily mucosal infections (e.g., tracheobronchitis, pneumonias, urinary tract infections), but also more serious systemic bacterial infections. More recently, opportunistic bacterial, viral (including HBV reactivation), and fungal infections have become more prominent with the increasing use of T cell-depleting agents such as purine nucleoside analogs and the anti-CD52 monoclonal antibody alemtuzumab. Anti-CD20 antibodies contribute to infection risk by causing B cell depletion, hypogammaglobulinemia, and worsening neutropenia. Tyrosine kinase inhibitors likely cause increased infection risk through a variety of mechanisms, including B cell dysfunction, worsening neutropenia, and, possibly, due to inhibitory effects on IL-2–inducible T cell kinase (in the case of ibrutinib); idelalisib, in particular, appears to be associated with a heightened risk of opportunistic infections including disseminated herpes virus infections and *P*. *jirovecii* pneumonia [40, 41].

Diarrhea of any grade was reported in 19.8% of patients with two (2.2%) having reported a grade 3 event, one of which required a brief treatment interruption and subsequent dose reduction. Considering the incidence of diarrhea reported among ibrutinib-treated patients in this disease setting (48 to 49%; grade ≥ 3, 2 to 4%) [3, 9], the lower incidence reported in the current study may be a consequence of less zanubrutinib-associated EGFR inhibition. An important component of the safety evaluation for this study was the assessment of AEs of interest, identified on the basis of the clinical experience with other BTK inhibitors. Hypertension was reported in nine (9.9%) patients (2 with grade 3 events), 6 of whom had a history of hypertension. Only 2 patients with treatment-emergent hypertension required new antihypertensive therapy and no patient required zanubrutinib treatment modification for hypertension management. The incidence of hypertension among ibrutinib-treated patients in this disease setting has been 18 to 27% (grade ≥ 3, 5 to 13%) after a median follow-up of 20.9 to 27.6 months [3, 38]. Over longer ibrutinib treatment intervals (median, 39 months), the incidence of grade ≥ 3 hypertension in relapsed/refractory patients has been reported at 25%, which may indicate increasing incidence with longer exposures [12]. Importantly, there were no occurrences of atrial fibrillation/flutter in the current study compared with grade ≥ 3 atrial fibrillation reported in 4 to 8% of ibrutinib-treated patients with relapsed/refractory CLL/SLL [9, 12]. This finding is consistent with results from the BGB-3111-AU-003 study of zanubrutinib in a cohort of 94 CLL/SLL patients treated at trial sites outside of China in which only one occurrence of atrial fibrillation in a patient with a history of hypertension and hyperlipidemia was reported [15]. Risk factors for atrial fibrillation were generally uncommon among patients in the current study with the notable exceptions of a history of hypertension (in 29% of patients) and diabetes mellitus (in 18%) (Supplemental Table 5). Other factors that may account for the absence of atrial fibrillation in the current study include the younger patient population (as previously noted), as well as epidemiologic evidence for a lower age-standardized incidence of atrial fibrillation among elderly Chinese compared with Western populations [42], although it is noteworthy that in a recent study of ibrutinib in mostly Chinese R/R CLL/SLL patients (median age, 65 years), 5.8% of patients reported treatment-emergent atrial fibrillation [39]. While minor mucocutaneous bleeding events were relatively common, only 2 (2.2%) patients reported major bleeding (gastrointestinal hemorrhage in the setting of newly diagnosed colon cancer and post-traumatic right thalamic hemorrhage). Nineteen (20.9%) patients received medications with anti-thrombotic properties while on study; 17 received analgesic/anti-inflammatory medications (e.g., NSAIDs, aspirin), 1 received enoxaparin sodium alone, and 1 received calcium heparin and clopidogrel. All exposures were short term, lasting 1–5 days; neither of the patients with major hemorrhage were receiving concurrent antithrombotic agents. By comparison, 1 to 9% of ibrutinib-treated patients reported major hemorrhage (including intracranial hemorrhage) [9, 38]. The incidence of grade ≥ 3 neutropenia and thrombocytopenia reported in the current study was high in comparison with that reported in populations of R/R CLL/SLL patients enrolled to ibrutinib clinical studies outside of China [3, 9, 36]. Despite indications that patients in the RESONATE trial [9] were more heavily pretreated than patients in the current study, there was less myelosuppression in the former study. In particular, grade ≥ 3 neutropenia was reported in 20% of ibrutinib-treated RESONATE patients in extended follow-up (median 19 months) [36] vs 44% of patients in our study after a median follow-up of 15.1 months. Since neutropenia is an on-target toxicity of BTK inhibitors and ibrutinib and zanubrutinib inhibit BTK via the same mechanism, the higher incidence of grade ≥ 3 neutropenia among zanubrutinib recipients can be attributed, at least in part, to its greater bioavailability as measured by relative plasma exposures for the two agents [15]. Predictably, the relatively low incidence of grade ≥ 3 neutropenia in a cohort of treatment-naive CLL/SLL patients treated with zanubrutinib (10%) [43] is consistent with a role for prior myelotoxic agent and anti-CD20 exposures in the pathogenesis of severe neutropenia reported herein (Table 1). The frequency and severity of other AEs of interest (e.g., mild or moderate mucocutaneous bleeding) were generally consistent with those reported in ongoing clinical studies of zanubrutinib in other B cell malignancies [44]. Other less serious but potentially bothersome toxicities reported with ibrutinib, including arthralgias (18%), headache (15%), vomiting (14%), myalgias (11%), muscles spasms (13%), and blurred vision (10%) [9], were uncommon in the current study (≤ 6%).

A limitation of the current study is the single-arm design, which limits both efficacy and safety comparisons with other BTK inhibitors. Two ongoing randomized studies of zanubrutinib versus ibrutinib, one in treatment-naïve and one in relapsed/refractory CLL/SLL patients (NCT03053440 and NCT03734016), aim to determine whether consistent, continuous BTK blockade with a more selective inhibitor results in fewer off-target effects and translates into improvements in disease control.

## Conclusion

This phase 2 study of patients with relapsed/refractory CLL/SLL recruited from trial sites in China demonstrated that twice-daily administration of zanubrutinib resulted in a high rate of durable responses. As a selective BTK inhibitor with favorable pharmacokinetic and pharmacodynamic properties, zanubrutinib offers the potential for improved safety and tolerability over existing treatment options and thereby potentially confers a favorable benefit-risk profile for patients with relapsed/refractory CLL/SLL.

## Supplementary information

**Additional file 1: Supplemental Table 1.** Adverse Events of Interest: Categories and Corresponding Search Criteria. **Supplemental Table 2.** Treatment-Emergent Adverse Events Leading to Zanubrutinib Discontinuation or Dose Reduction. **Supplemental Table 3.** Adverse Events of Special Interest by Category. **Supplemental Table 4.** Co-administration of Moderate or Strong CYP3A Inhibitors in Study BGB-3111-205. **Supplemental Table 5.** Risk Factors for Atrial Fibrillation Among Patients Enrolled to Study BGB-3111-205. **Supplemental Figure 1.** Changes in Absolute Lymphocyte Counts Over Time

## Data Availability

Additional data are provided in the data supplement available online. Individual participant data will be shared upon review/request after global regulatory approval of zanubrutinib for the treatment of CLL/SLL.

## Abbreviations

AE: Adverse event
BTK: Bruton tyrosine kinase
CLL: Chronic lymphocytic leukemia
CMQ: Company MedDRA Query
CR: Complete response
CRi: Complete response with incomplete bone marrow recovery
DOR: Duration of response
ECOG PS: Eastern Cooperative Oncology Group performance status
EGFR: Epidermal growth factor receptor
G-CSF: Granulocyte colony-stimulating factor
HBV: Hepatitis B virus
HCV: Hepatitis C virus
IGHV: Immunoglobulin heavy-chain variable region
IRC: Independent review committee
ITK: Interleukin-2-inducible T cell kinase
JAK3: Janus-associated kinase 3
LDH: Lactate dehydrogenase
LDi: Longest diameter
MedDRA: Medical Dictionary for Regulatory Activities
NA: Not applicable
NE: Not estimable
nPR: Nodular partial response
NSAIDs: Nonsteroidal anti-inflammatory drugs
ORR: Overall response rate
PD: Progressive disease
PFS: Progression-free survival
PR: Partial response
PR-L: Partial response with lymphocytosis
PT: Preferred term
R/R: Relapsed/refractory
SD: Stable disease
SLL: Small lymphocytic lymphoma
SMQ: Standardized MedDRA Query
SOC: System organ class
SPD: Sum of perpendicular diameters
TEAE: Treatment-emergent adverse event
TEC: Tyrosine kinase expressed in hepatocellular carcinoma
*TP53*: Tumor protein 53
ULN: Upper limit of normal
