# Supplementary material for: Treatment of relapsed/refractory chronic lymphocytic leukemia/small lymphocytic lymphoma with the BTK inhibitor zanubrutinib: phase 2, single-arm, multicenter study
Source: J Hematol Oncol. 2020 May 11;13:48. doi: 10.1186/s13045-020-00884-4 (PMC7216400; doi:10.1186/s13045-020-00884-4)
Supplement: Supplementary file 1 — Additional file 1: Supplemental Table 1. Adverse Events of Interest: Categories and Corresponding Search Criteria. Supplemental Table 2. Treatment-Emergent Adverse Events Leading to Zanubrutinib Discontinuation or Dose Reduction. Supplemental Table 3. Adverse Events of Special Interest by Category. Supplemental Table 4. Co-administration of Moderate or Strong CYP3A Inhibitors in Study BGB-3111-205. Supplemental Table 5. Risk Factors for Atrial Fibrillation Among Patients Enrolled to Study BGB-3111-205. Supplemental Figure 1. Changes in Absolute Lymphocyte Counts Over Time [file 13045_2020_884_MOESM1_ESM.docx]

**Treatment of Relapsed/Refractory** **Chronic Lymphocytic Leukemia/Small Lymphocytic Lymphoma With the BTK Inhibitor Zanubrutinib: Phase 2, Single-Arm, Multicenter Study**

Wei Xu, MD,^1^ Shenmiao Yang, MD,^2^ Keshu Zhou, MD,^3^ Ling Pan, MD,^4^ Zengjun Li, MD,^5^ Jianfeng Zhou, MD,^6^ Sujun Gao, MD,^7^ Daobin Zhou, MD,^8^ Jianda Hu, MD,^9^ Ru Feng, MD,^10^ Haiwen Huang, MD,^11^ Meng Ji, MD,^12^ Haiyi Guo, MD,^12^ Jane Huang, MD,^12^ William Novotny MD,^12^ Shibao Feng, PhD,^12^ Jianyong Li, MD^1,*^

**Supplementary Material**

**Supplemental Table 1. Adverse Events of Interest: Categories and Corresponding Search Criteria**

| AE of Interest Category | Search Criteria |
| --- | --- |
| Hemorrhage (including minor bleeding, such as contusion and petechiae) | Hemorrhage terms (excluding laboratory terms) (SMQ) Narrow |
| Major hemorrhage: Defined as serious or grade ≥3 bleeding at any site, or central nervous system bleeding of any grade | Major hemorrhage:   - Subdural hematoma PT, subdural hemorrhage PT - All hemorrhage PTs if AE SOC is “nervous system disorders” or - Serious or grade ≥3 hemorrhage PT if AE SOC is not “nervous system disorders” |
| Atrial fibrillation and/or flutter | Atrial fibrillation PT, atrial flutter PT |
| Hypertension | Hypertension (SMQ) narrow |
| Second primary malignancies  Skin cancers | Malignant tumors (SMQ) narrow  Subcategory – Skin malignant tumors (SMQ) narrow |
| Tumor lysis syndrome | Tumor lysis syndrome (SMQ) narrow |
| Infection  Opportunistic infections | Infections: Infections and infestations SOC  Subcategory – Opportunistic infections: opportunistic infections (CMQ) |
| Neutropenia | Neutropenia PT, neutrophil count decreased PT, febrile neutropenia PT, agranulocytosis PT, neutropenic infection PT, neutropenic sepsis PT |
| Thrombocytopenia | Thrombocytopenia PT, platelet count decreased PT |
| Anemia | Anemia PT, hemoglobin decreased PT |

AE, adverse event; CMQ, Company MedDRA Query; MedDRA, Medical Dictionary for Regulatory Activities; PT, preferred term; SMQ, Standardized MedDRA Query; SOC, system organ class

**Supplemental Table 2. Treatment-Emergent Adverse Events Leading to Zanubrutinib Discontinuation or Dose Reduction**

|  | **N (%)** |
| --- | --- |
| **TEAEs Leading to Zanubrutinib Discontinuation** | |
| Patients with at least 1 AE leading to zanubrutinib discontinuation | 8 (8.8) |
| Lung infection; cardiac failure; respiratory failure^a^ | 1 (1.1) |
| Pneumonia; infected dermatitis | 1 (1.1) |
| Hepatitis B reactivation | 1 (1.1) |
| Intracranial hemorrhage^b^ | 1 (1.1) |
| Lung infection | 1 (1.1) |
| Cardiopulmonary failure^a^ | 1 (1.1) |
| Breast cancer | 1 (1.1) |
| Colon cancer; gastrointestinal hemorrhage; anemia | 1 (1.1) |
| **TEAEs Leading to Zanubrutinib Dose Reduction** | |
| Patients with at least 1 AE leading to dose reduction^c^ | 7 (7.7) |
| Diarrhea | 1 |
| Lung infection | 3 |
| Hepatitis B reactivation | 2 |
| Hypokalemia | 1 |
| Hyponatremia | 1 |

^a^Grade 5 events (see text).

^b^Post-traumatic right thalamic hemorrhage on study day 369.

^c^One patient had two dose reductions (both for lung infection); the remaining patients had one dose reduction.

TEAE, treatment-emergent adverse event.

**Supplemental Table 3.** **Adverse Events of Special Interest by Category**

| **Category** | **Grades 1-2** | **Grade 3** | **Grade 4** |
| --- | --- | --- | --- |
|  | **n (%)** | | |
| Hematologic | | | |
| Neutropenia | 23 (25.3) | 34 (37.4) | 6 (6.6) |
| Thrombocytopenia | 24 (26.4) | 13 (14.3) | 1 (1.1) |
| Anemia | 19 (20.9) | 8 (8.8) | 0 |
| Hemorrhage (including minor cutaneous bleeding) | 56 (61.6) | 1 (1.1) | 0 |
| Major hemorrhage^a^ | 1 (1.1) | 1 (1.1) | 0 |
| Nonhematologic | | | |
| Infections | 45 (49.5) | 34 (37.4) | 0^b^ |
| Opportunistic infections | 1 (1.1)^c^ | 0 | 0 |
| Atrial fibrillation/flutter | 0 | 0 | 0 |
| Hypertension | 7 (7.7) | 2 (2.2) | 0 |
| Second primary malignancies | 0 | 2 (2.2)^d^ | 0 |
| Tumor lysis syndrome | 0 | 0 | 0 |

^a^Defined as serious or grade ≥3 bleeding at any site or central nervous system bleeding of any grade. One patient had a grade 2 post-traumatic right thalamic hemorrhage; one patient had a grade 3 gastrointestinal hemorrhage in the setting of newly diagnosed colon cancer (see text).

^b^One patient had a grade 5 lung infection (see text).

^c^One patient had herpes simplex.

^d^One patient developed new-onset breast cancer; one patient had newly diagnosed colon cancer (see text).

**Supplemental Table 4.** **Co-administration of Moderate or Strong CYP3A Inhibitors in Study BGB-3111-205**

| **CYP3A Inhibitor** | **No. (Percent) of CYP3A Inhibitor Exposed Patients (n=7) ^a^** | **No. of Consecutive Exposure Days to CYP3A Inhibitor** | **Status of Zanubrutinib Treatment During CPY3A Inhibitor Therapy** | **Grade 3 and/or Serious Adverse Events during and after CYP3A Inhibitors ^d^** |
| --- | --- | --- | --- | --- |
| Fluconazole (moderate) | 4 (4.4%) | 1, 1, 22, 139 | Full dose (n=2 ^b, c^); Interrupted (n =1 )  Permanently discontinued (n=1) ^e^ | 1/4 ^c^ |
| Voriconazole (strong) | 4 (4.4%) | 1, 11, 12, 67 | Permanently discontinued (n=4) ^e^ | 0/4 |

^a^ Three patients were treated with voriconazole alone, 3 with fluconazole alone and 1 with a combination of fluconazole (for 1 day) followed by voriconazole (for 11 days).

^b^ One patient received only 1 dose of fluconazole while receiving full dose zanbrutinib, without incident.
^c^ One patient presented with grade 4 neutropenia on study day 283 after having completed an extensive prior exposure to fluconazole lasting 139 days while concurrently receiving full dose zanubrutinib.

^d^ Observation time for adverse events was from the time of antimycotic therapy initiation until 30 days after completion.

^e^ Zanubrutinib was interrupted prior to administration of the CYP3A inhibitor and not re-started.

**Supplemental Table 5. Risk Factors for Atrial Fibrillation Among Patients Enrolled to Study BGB-3111-205**

| **Risk Factor** | **Patients, n (%)** |
| --- | --- |
| History of atrial fibrillation | 1 (1) |
| Hypertension | 26 (29) |
| Diabetes mellitus | 16 (18) |
| Coronary artery disease | 2 (2) |
| Valvular heart disease | 2 (2) |
| Dyslipidemias | |
| Hypertriglyceridemia | 27 (30) |
| Hypercholesterolemia | 8 (9) |

**Supplemental Figure 1. Changes in Absolute Lymphocyte Counts Over Time**


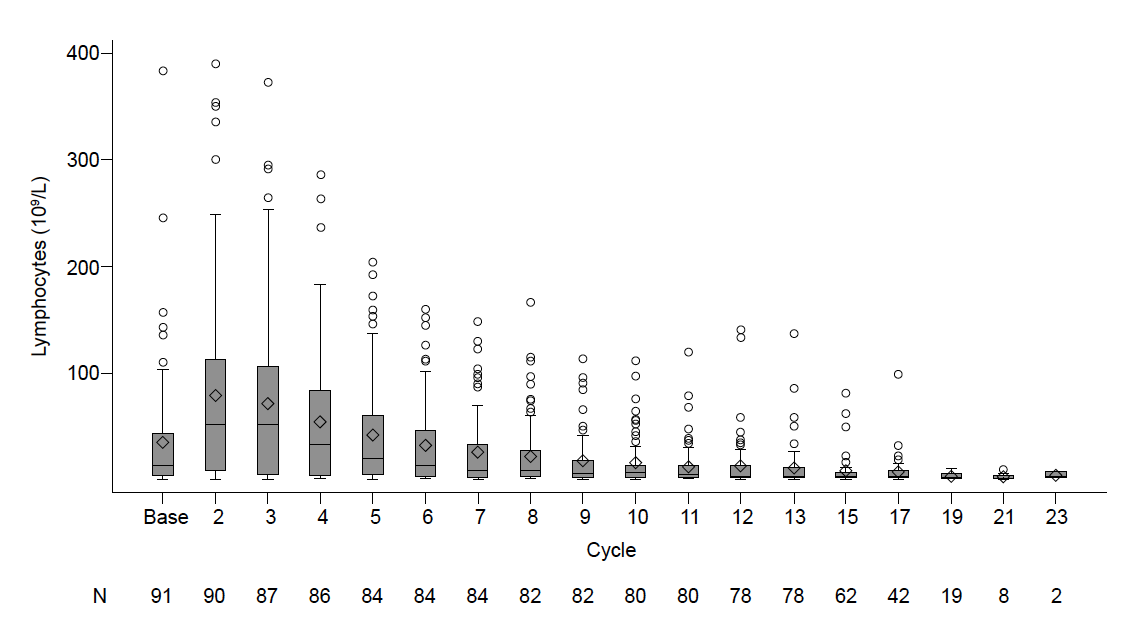


Note: The boxes represent the 25th and 75th percentile, the whiskers represent the minimum and maximum values (excluding outliers). Outliers (open circles) were defined as values beyond 1.5 times the interquartile range. The diamonds represent the mean; the horizontal lines represent the median. Number of patients with observed values at each visit are displayed at the bottom of the figure.
